# Maternal practices and perceptions of child body mass status explain child energy expenditure behaviors and body mass

**DOI:** 10.1007/s10865-020-00138-1

**Published:** 2020-01-31

**Authors:** Monika Boberska, Karolina Zarychta, Nina Knoll, Jan Keller, Diana Hilda Hohl, Karolina Horodyska, Magdalena Kruk, Aleksandra Luszczynska

**Affiliations:** 1 Faculty of Psychology, SWPS University of Social Sciences and Humanities, 30b Ostrowskiego St, 53238 Wroclaw, Poland; 2 Department of Education and Psychology, Freie Universität Berlin, Habelschwerdter Allee 45, 14195 Berlin, Germany; 3 Trauma, Health and Hazards Center, University of Colorado, 1861 Austin Bluffs Pkwy, Colorado Springs, CO 80933-7150 USA

**Keywords:** BMI z-score, Physical activity, Sedentary behavior, Screen use, Child, Mother

## Abstract

This study investigated whether maternal perceptions of child body mass status would predict child body mass index (BMI) z-score via two sets of sequential mediators: (1) four maternal practices promoting child energy expenditure and (2) children’s energy expenditure behaviors. The data of *N* = 729 mother–child dyads were collected at baseline [T1; *n* = 495 at 7- to 8-month follow-up (T2)]. Mothers reported perceptions of child body mass status and maternal practices (T1); children reported sedentary screen use and physical activity (T1, T2). Child body mass was assessed objectively (T1, T2). Higher stimulation to be active (T1) was related to a lower child BMI z-score (T2) via higher levels of child physical activity (T2). Higher levels of monitoring of screen use (T1) were associated with higher child BMI z-score (T2) via lower levels of child physical activity (T2). Encouraging parents to stimulate their children to be active may be beneficial for children’s weight maintenance.

**Electronic supplementary material:**

The online version of this article (10.1007/s10865-020-00138-1) contains supplementary material, which is available to authorized users.

## Background

The global age-standardized prevalence of obesity in children and adolescents increased from 0.7% in 1975 to 5.6% in 2016 in girls and from 0.9% (1975) to 7.8% (2016) in boys (World Health Organization; WHO, 2016). Energy expenditure behaviors, such as physical activity (PA) and sedentary screen use in leisure time (referred to as “screen use” for brevity), are the key behavioral determinants of overweight and obesity in children (WHO, 2016). Across European countries, less than 50% of children younger than 12 years old comply with recommended levels of physical activity (Van Hecke et al., 2016). Screen use is a type of sedentary behavior (SB) that involves the use of screen-based appliances, such as watching television, using a computer, sedentary socializing using mobile phones or tablets, or playing electronic games (Biddle, Pearson, & Salmon, 2018). It is among the most frequently performed SBs in childhood (Arundell, Fletcher, Salmon, Veitch, & Hinkley, 2016). Children who are overweight tend to have higher levels of screen use time than children with a normal body mass (for review see Biddle et al., 2018).

Theoretical models explaining energy expenditure behaviors (e.g., physical activity or screen use) among children typically suggest that maternal behaviors and perceptions are the key determinants of child behaviors and childhood obesity (Gubbels et al., 2011). Social-ecological models indicate that parental perceptions, parental practices, and child behaviors (including physical activity and screen use) influence childhood obesity (Davison & Birch, 2001; Sallis et al., 2006). For example, the model of energy balance-related parenting (Gubbels et al., 2011) suggests that parental practices have direct effects on energy expenditure behaviors and childhood obesity. Similarly, the framework proposed by Biddle et al. (2018) indicates that social factors (such as parental practices or parental obesity-related perceptions) may indirectly predict obesity via behavioral mediators (e.g., physical activity or screen use).

According to the model of energy balance-related parenting, four parental practices are directly related to energy expenditure behaviors (e.g., physical activity and screen use) and indirectly related to childhood obesity (Gubbels et al., 2011). *Stimulation to be active* is used by parents to motivate their children to be more active, for instance, commit to walking and cycling (Gubbels et al., 2011). *Restrictions of screen use* are applied by parents to ascertain that children do not spend too much time on sedentary screen use, with parents providing clear verbal messages to enforce time limits for screen use (Gubbels et al., 2011). While restriction strategies usually involve verbal instructions, monitoring refers to parental engagement in observing and tracking children’s behaviors. In particular, *monitoring of PA* refers to keeping track of child engagement in physical activity, while *monitoring of screen use* refers to keeping track of time children spend watching television or playing computer games. Although these four parenting strategies refer to either physical activity or sedentary behavior, each of them is assumed to influence both physical activity and sedentary behavior (Gubbels et al., 2011).

To date, the majority of research investigating the associations between parental practices, child behaviors, and child body mass has been cross-sectional. These types of studies have confirmed associations between physical activity in children younger than 12 years old and parental practices, such as stimulation to be active or monitoring of PA (Edwardson & Gorely, 2010). A limited number of studies investigating longitudinal associations among parental strategies, child sedentary behavior and body mass have yielded mixed findings. For example, one longitudinal study with parents of 5- to 7-year-old children found that parental reports of restrictions of screen use predicted higher levels of child screen use, lower levels of child physical activity, and higher levels of child body mass (Sleddens, Gubbels, Kremers, van der Plas, & Thijs, 2017). Parental stimulation to be active predicted child physical activity but was unrelated to child screen use or body mass, whereas monitoring of PA was unrelated to physical activity, screen use, or body mass in children. On the other hand, a study enrolling parent–child dyads and using objective measures of body fat in children indicated that parental restrictions of screen use (reported by children) predicted lower child body fat assessed 7- to 8- months later (Boberska et al., 2019). To date, the majority of research focusing on the link between parental practices and energy expenditure behaviors in children has used a cross-sectional design and self-reports of children or parents only, but not both (e.g., Jago, Wood, Zahra, & Thompson, 2015; Lloyd, Lubans, Plotnikoff, Collins, & Morgan,^2014^). Consequently, the dyadic perspective has received limited attention.

As the prevalence of childhood obesity is growing, parents must handle the difficult task of determining whether their child is overweight (Young et al., 2010). There is evidence of a tendency among parents (especially among mothers) to perceive their child as having normal body mass, regardless of their child’s actual body mass (Crawford, Timperio, Telford, & Salmon, 2006; Merema et al., 2015; Webber, Hill, Cooke, Carnell, & Wardle, 2010). Parents who perceive their child as having a higher than normal body mass are more likely to introduce changes in child nutrition and physical activity than parents who perceive their child as having a normal body mass status (Merema et al., 2015; Sylvetsky-Meni, Gillepsie, Hardy, & Welsh, 2015). Research showed that parents who perceive their children as being overweight were more likely to be in the preparatory or action stages of behavior change, ready to engage in behaviors to help their children to lose weight, than were parents who did not perceive their children as overweight (Rhee, Lago, Arscott-Mills, Mehta, & Davis, 2005).

Maternal perceptions of child body mass status are well-recognized predictors of maternal practices related to child energy intake (Webber et al., 2010). Mothers who perceive their children as having normal body mass often encourage their 5- to 7-year-old children to eat more than mothers who perceived their children as being overweight do (Yilmaz, Erkorkmaz, Ozcetin, & Karaaslan, 2013). Parents who perceive their children as being overweight are more likely to restrict unhealthy food intake (Wehrly, Bonilla, Perez, & Liew, 2014). These perceptions of child body mass status often have more influence on parental practices regulating child energy intake than on the actual body mass of a child (Merema et al., 2015). Aside from body mass, less is known about the associations between parental perceptions of child body mass status and parental practices promoting energy expenditure behaviors, such as physical activity or screen use reduction.

Studies investigating the role of parental perceptions of child body mass and parental practices aiming at promoting child energy expenditure have usually focused on maternal predictors (e.g., 93–98% mothers; Seburg et al., 2014, Sleddens et al., 2017; 100% mothers; Maynard et al., 2003). As the existing evidence most often refers to mothers, it seems prudent to investigate the associations between parental predictors of child energy expenditure behaviors in a sample involving mothers only.

## Aims of the study

Using a prospective design and dyadic mother–child data, this study aimed to investigate the relationship between maternal perceptions of child body mass status and child BMI z-score via two sets of sequential mediators: (1) four maternal practices and (2) two child behaviors, namely, screen use and physical activity. First, we hypothesized that maternal perceptions of child body mass status (time 1 [T1]) would indirectly predict child screen use and physical activity (time 2 [T2]), with four maternal practices aiming to increase child energy expenditure (T1) acting as parallel mediators. The four maternal practices were restrictions of screen use, stimulation to be active, monitoring of screen use, and monitoring of PA. Second, we hypothesized that the four maternal practices (T1) would indirectly predict child BMI z-score (T2) via screen use and physical activity in children (T2). Additionally, we explored direct associations between maternal perceptions of child body mass status and maternal practices, screen use, physical activity, and BMI z-score in children.

## Methods

### Participants

This study was part of a larger project investigating healthy lifestyle in parent–child dyads (see Horodyska et al., 2017). Mothers and their 5- to 11-year-old children (*N* = 729 dyads, *N* = 1,458 individuals) participated in the baseline measurement (T1), whereas *n* = 495 mother–child dyads provided their responses at T2 (7–8 months after T1).

Attrition analyses indicated that mothers who completed T1 and T2 did not differ from mothers who dropped out in terms of parental strategies or socioeconomic status but differed in terms of age (mothers who dropped out were younger; see Supplement 1). Children who completed T1 and T2 did not differ from children who dropped out in terms of age, gender, or physical activity. Those who dropped out had a higher BMI z-score and reported more screen use (Supplement 1).

Mothers or female legal guardians (henceforth referred to as “mothers”) who indicated that they were the main caregivers in terms of time spent with their child and organizing child PA were invited to participate. Dyads with children with impairments and physical disabilities resulting in major movement restrictions (e.g., cerebral palsy) were excluded from the study. Regarding younger children (aged 5–7 years old), only those who either had already attended primary schools or reached school readiness (required to start first grade) were included.

### Procedure

Prior to the study from which the data for this analysis was obtained, a pilot study with *N* = 18 children (aged 5–11 years old) was conducted to check participants’ understanding of the items assessing screen use and physical activity. Children were asked to explain the instructions and the items in their own words and to indicate any phrases they did not understand/were unsure of. The pilot study indicated that, using provided instructions and the items, children were able to correctly classify their behaviors referring to screen use and mild, moderate, and vigorous physical activity.

Data were collected between 2011 and 2015. Potential respondents were approached in 26 locations of 8 administrative regions of Poland. To represent economic diversity, the data were obtained from locations in the regions characterized as lower in economic development (23% of locations), medium in economic development (50%), and higher in economic development (27%). Data were collected in schools and general practitioners’ offices. In each potential location, the research team visited primary schools providing education for children aged 5–11. The team also visited nurses’/general practitioners’ offices (conducting routine check-ups among children aged 5–11 years old) and discussed the possibility of data collection. Two schools (out of 27 approached) and two practitioners’ offices (out of 12 approached) did not agree to contribute to the collection of data.

The potential participants were recruited during the parent-teacher meetings, school classes, and check-up visits at the general practitioners’ offices. Mothers and children were given information about the study participation schedule and the aims of the study, and they were told that their participation would be kept confidential. Informed consent was collected from mothers (concerning their own and their child’s participation) and written assent was obtained from children. Personal codes were used to ensure confidentiality.

At T1, researchers conducted oral interviews with children aged 5–8 and those who declared that they were not good at reading or writing. Older children (aged 9–11) completed a questionnaire. Mothers provided self-report data. Data from mothers and children were collected separately. After the questionnaires/interviews were completed, child body weight and height were measured. At T2, study personnel revisited the study locations three times after contacting mothers by phone. Data were collected at two time points: the beginning of the school year (T1) and at the end of the school year (T2). Thus, dropout due to school change after the completion of a school year was limited.

The study was approved by the Ethics Committee at the first author’s institution. The respective dataset is available at https://osf.io/r8qmg/.

### Measures

Descriptive statistics for all measures are provided in Table 1.

**Table 1:** Correlations and descriptive statistics for the study variables

	2	3	4	5	6	7	8	9	10	11	12	13	14	15	16	*M (SD)*
1. Perceptions of child body mass (M, T1)	.05	.03	− .01	.06	− .03	.06	.00	.01	**.51**	**.50**	.07	.06	− .03	.04	.04	3.00 (0.55)
2. Restrictions of screen use (M, T1)		**.51**	**.39**	**.44**	.04	.07	.03	.06	.05	.03	.05	.04	.00	− .05	− **.14**	2.75 (0.74)
3. Monitoring of screen use (M, T1)			**.45**	**.45**	.01	.02	.02	− .01	− .03	− .02	**.15**	**.13**	.01	− **.10**	− .09	4.02 (0.93)
4. Monitoring of PA (M, T1)				**.45**	− .01	.04	.07	.08	− .03	− .02	**.13**	.05	.01	− .05	− .04	3.38 (0.65)
5. Stimulation to be active (M, T1)					.06	**.14**	**.14**	.**17**	.01	.00	.07	.08	.05	− .04	− **.11**	3.10 (0.63)
6. Screen use (Ch, T1)						**.43**	.01	.01	.04	.05	− **.13**	− .01	− .09	− .04	− .08	3.40 (3.10)
7. Screen use (Ch, T2)							.03	.03	.09	.07	− **.19**	− .03	− .07	− .03	− **.14**	3.33 (2.76)
8. Physical activity (Ch, T1)								.**19**	− .02	.00	.06	.08	.00	.05	− **.13**	55.61 (29.86)
9. Physical activity (Ch, T2)									.05	.00	− .01	.00	.07	**.11**	− .09	57.47 (26.52)
10. BMI z-score (Ch, T1)										**.94**	− .06	.03	− **.12**	**.10**	− .04	0.45 (1.24)
11. BMI z-score (Ch, T2)											− .02	.03	− **.11**	.08	− .04	0.32 (1.22)
12. Education (M, T1)												**.29**	**.16**	− .02	− .04	3.67 (1.28)
13. SES (M, T1)													.06	− .03	− .05	3.26 (0.80)
14. Age (M, T1)														**.18**	.00	36.10 (5.64)
15. Age (Ch, T1)															− .02	8.42 (1.35)
16. Gender (Ch, T1)																1.53 (0.50)

Correlation coefficient values at *r* > .07 were significant at *p* < .05. Correlation coefficient values at *r* > .10 were significant at *p* < .01. Correlation coefficient values at *r* > .13 were significant at *p* < .001. *M* mother; *Ch* child; *T1* time 1 (baseline); *T2* time 2 (7- to 8-month follow-up). Perceptions of child body mass = maternal perceptions of child body mass status; restrictions of screen use = maternal restrictions of sedentary screen use behaviors; screen use = sedentary screen use behaviors in children; *PA* physical activity; SES = maternal perceived economic status. Significant coefficients are marked in bold

Maternal perceptions of child body mass status (maternal perceptions of child body mass) were assessed at T1 with a one-item measure developed by Czajka and Kolodziej (2015): “How would you describe your child body mass?” The responses were given on a 5-item response scale (1—*significantly underweight*, 2—*slightly underweight*, 3—*normal body mass*, 4—*slightly overweight*, 5—*significantly overweight*). Using a one-item measure to assess this construct is a standard approach that is applied in numerous studies (e.g., Gerards et al., 2014, Robinson & Sutin, 2016).

The four types of maternal practices were measured with subscales developed to measure maternal practices associated with physical activity and sedentary behaviors among children aged 5–7 years old (Gubbels et al., 2011). Mothers were instructed to refer to the past week (previous 7 days).

Maternal restrictions of screen use (restrictions of screen use) were measured at T1 with 4 items from Gubbels et al. (2011), e.g., “I intentionally keep my child away from sitting and watching TV, playing computer games, using tablets/phones, etc.” The responses were given on a 4-item response scale ranging from 1 (*definitely not*) to 4 (*exactly true*). The internal consistency of the scale was acceptable, with *α* = .80.

Maternal stimulation to be active (T1) was assessed with 3 items from the scale by Gubbels et al. (2011), e.g., “If my child says’I don’t feel like walking or bicycling’, I try to get him/her to do this anyway”. The responses were given on a 4-item response scale ranging from 1 (*definitely not*) to 4 (*exactly true*). The internal consistency of the 3-item measure was acceptable, with *α* = .73.

Maternal monitoring of screen use (T1) was measured with 2 items from Gubbels et al. (2011), e.g., “How much do you keep track of the amount of television your child watches?” The responses were given on a 5-item response scale ranging from 1 (*never*) to 5 (*always*). The internal consistency of the 2-item measure was acceptable, with Spearman’s ρ = .86.

Maternal monitoring of PA (T1) was measured with one item from the scale by Gubbels et al. (2011): “How much do you keep track of the amount of physical activity your child has?” The responses were given on a 5-item response scale from 1 (*never)* to 5 (*always*).

Child screen use in leisure time was measured at T1 and T2 using two items adapted from Maher, Mire, Harrington, Staiano, and Katzmarzyk, (2013). Children were instructed to consider screen use while sitting or reclining and were asked to refer to recreational activities performed during the previous week (previous 7 days). Examples of screen use during sitting or reclining were provided in verbal instruction at the beginning of the interview or questionnaire. Next, children were asked about their typical day: “How many hours per day do you spend on watching TV?” and “How many hours per day do you spend sitting and playing computer games, video games (including console games), using tablets, etc.?” An open-ended response format (number of hours per day) was used instead of the original 7-point response scale (Katzmarzyk et al., 2013; Maher et al., 2013). The sum scores of the two items were calculated. The internal consistency of the 2-item measure was acceptable, with ρ = .58 (T1) and ρ = .71 (T2).

Children’s self-reported physical activity was measured at T1 and T2, with three items derived from a self-report physical activity questionnaire by Godin and Shephard (1985). The validity and reliability of this questionnaire was found to be acceptable in studies involving children aged 7–15 (Koo & Rohan, 1999). At the beginning of the interview or questionnaire, verbal instructions were provided to clarify the differences between mild physical activity, moderate physical activity, and vigorous physical activity, with a reference to heartbeat, sweating, and ability to talk while exercising, followed by examples of mild-intensity exercises, moderate-intensity exercises and vigorous-intensity exercises. Children were asked to report how often (frequency per week) they exercise for more than 15 min during their free time (Godin & Shephard, 1985). The first item refers to “strenuous exercise (heart beats rapidly), e.g., running, jogging, hockey, soccer, basketball, judo, roller skating, vigorous swimming, vigorous long-distance bicycling”. The second item refers to “moderate exercise (not exhausting), e.g., fast walking, easy bicycling, easy swimming, dancing”. The third item refers to mild exercise, e.g., easy walking. To obtain a total metabolic equivalent (MET) score, the vigorous score was multiplied by 9, the moderate score was multiplied by 5, and the mild score was multiplied by 3. The scores were summed up (Godin & Shephard, 1985). The reliability of this measure was low, with α = .51 (T1) and α = .53 (T2).

The body weight and height of children (T1 and T2) were measured with certified body weight floor scales (BF-100 and BF-25; Beurer, Germany, measurement error < 5%) and medically approved telescopic height-measuring rods. Child BMI z-score values (T1 and T2) were calculated using child weight (kg) and height (m) and were based on WHO growth references, using the SPSS macro provided by the WHO (de Onis et al., 2007).

T1 data collection accounted for sociodemographic variables: child gender, maternal and child age, maternal education and perceived economic status. Maternal education was measured with a 5-point scale (primary, uncompleted secondary/vocational, secondary, ≤ 3 years of higher education, ≥ 5 years of higher education). Perceived economic status was assessed with one item, namely, “Compared to the average economic situation of a family in this country, how would you rate the economic situation of your family?”, with responses ranging from 1 (*far below average*) to 5 (*far above average*).

### Data analysis

To determine the sample size, the G*Power calculator (Faul, Erdfelder, Lang, & Buchner, 2007) was used. Assuming small effect sizes (*f*^2^ = .03) of relations between predictors and the child BMI z-score and considering that the analyses should account for potential confounders, we estimated that the sample should include at least 624 dyads at T1.

Manifest mediation analyses (Byrne, 2010) were conducted using maximum likelihood estimation procedures (IBM AMOS 25). Missing data (including data missing due to dropouts at T2) were accounted for by using the full information maximum likelihood procedure (FIML; Byrne, 2010). Little’s MCAR test indicated that the missing data patterns were systematic, Little’s χ^2^(299) = 379.73, *p* = .001. Several fit indices were applied to assess model-data fit. We used a cut-off point < .08 for the root mean square error of approximation (RMSEA) and standardized root mean residual (SRMR), as well as a cut-off point > .90 for the comparative fit index (CFI), the Tucker-Lewis index (TLI), the goodness-of-fit index (GFI), and the normed fit index (NFI) (Byrne, 2010). The significance of indirect effects was evaluated using bias-corrected (BC) bootstrapping with 10,000 resamples and calculating the 95% confidence interval (CI_BC_) (MacKinnon et al., 2004). Multivariate normality was checked with Mardia’s coefficient, with values of 54.49 indicating moderate non-normality.

The hypothesized mediation model assumes that in addition to forming direct associations with the dependent variables (DVs), the independent variables (IVs) may operate through the hypothesized mediators (MacKinnon, 2008): the IV predicts the mediators, which in turn predict the DV. Two types of effects may be tested: (1) the direct effects of the IV and of the mediators on the DV and (2) the indirect effects of the IV on the DV through the mediators (MacKinnon, 2008). The hypothesized model assumed (1) direct effects of the IV (maternal perception of child body mass at T1) and effects of maternal practices (T1) on the DV (child BMI z-score) and (2) indirect effects of the IV on the DV via two sets of mediators operating sequentially, namely, (a) maternal practices (T1) and (b) child screen use and physical activity (T2). As suggested for longitudinal mediation analysis (Roth & MacKinnon, 2012), adjustments were made for the baseline (T1) effects of the mediators and the DV in the model. Furthermore, analyses included control variables that were associated with the patterns of missing data (i.e., maternal age, child screen use, and child BMI z-score). The control variables included child screen use (T1), child physical activity (T1), child BMI z-score (T1), child age and gender, maternal age (T1), maternal education (T1), and maternal perceived economic status (T1). To conduct sensitivity analysis, the model was tested without control variables (except for child BMI z-score at T1, which was included in both analyzed models).

## Results

### Study sample characteristics

Mothers’ ages ranged between 23 and 68 years old (*M* = 36.1, *SD* = 5.64). The majority of mothers (66.3%) had a body mass index (BMI) of 18.50–25.0 kg/m^2^, indicating normal body mass; 2.4% had a BMI of less than 18.50 kg/m^2^; 24% of mothers had a BMI of 25.00–30.00 kg/m^2^, indicating overweight; and 7.3% had a BMI of more than 30.00 kg/m^2^, indicating obesity. The majority of mothers had either secondary education (27.6%) or higher education (40.2%); 59% of mothers reported full-time employment; and 59% reported that their perceived economic status was similar to the economic status of the average family in Poland. The majority of mothers (68%) lived in urban areas. All participants were white (for a full description of sociodemographic characteristics, see Supplement 1).

Children (53.1% girls) were 5–11 years old (*M* = 8.42, *SD* = 1.35); only 0.7% were 5 years old, 9.8% were 6 years old and 89.5% were 7–11 years old. Across the majority of study variables, there were no significant differences between younger children (aged ≤ 8 years old) and older children (aged ≥ 9 years old), except for higher child body mass, older maternal age, and less frequent use of maternal monitoring of screen use in older children (Supplementary Table 1).

The majority (66.5%) of children had normal body mass, 23.6% were overweight or obese, and 9.9% were underweight when applying the International Obesity Task Force Thresholds (Cole & Lobstein, 2012). Overall, 74.3% of mothers considered their child to have normal body mass, 12.8% indicated child overweight/obesity, and 12.9% identified their children to be underweight. In dyads with measured overweight/obese children, only 41.9% of mothers perceived their child as overweight/obese, 52.9% considered their child to have normal body mass, and 5.2% indicated that their child was underweight (see Supplementary Table 2). Only 0.7% of children met physical activity recommendations (WHO, 2018) of > 60 daily minutes of moderate-to-vigorous physical activity (0.8% among children with normal body mass; 0.6% among children with overweight/obesity; see Supplementary Table 2).

Comparisons of mother-daughter and mother-son dyads yielded several significant differences (see Supplement 1). Compared to mother-daughter dyads, mother-son dyads involved mothers’ more frequent restriction of their sons’ screen use, higher levels of monitoring their sons’ screen use, and sons’ higher level of stimulation to be active. Compared to girls, boys reported higher levels of physical activity and higher levels of screen use.

### Findings for the hypothesized model: indirect associations between maternal perceptions and child BMI z-score, mediated by maternal practices, child screen use, and physical activity

Bivariate analyses indicated that only one maternal practice, namely, stimulation to be active, was significantly associated with child energy expenditure behaviors (see Table 1). Screen use and physical activity were unrelated.

**Fig. 1:**
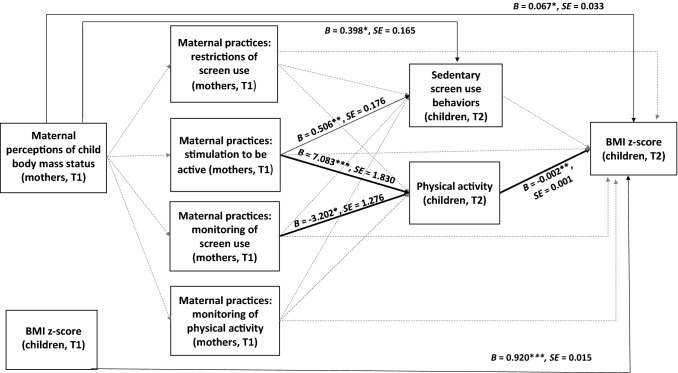
Results of the path analysis for the hypothesized mediator model for the total sample (*N* = 729 dyads). *T1* time 1 (baseline); *T2* time 2 (7- to 8-month follow-up). For clarity, the effects of the control variables which were included in the model (T1 variables: child screen use, physical activity and BMI z-score, child gender, maternal and child age, maternal education, and perceived economic status) are not displayed. The values of unstandardized path coefficients, *SE* and *p*-levels are displayed for significant coefficients only. Solid lines represent path coefficients which were significant. Bold solid lines represent significant indirect effects. Dashed lines represent path coefficients which were not significant. For clarity, the covariances were not displayed. Path and covariance coefficients are presented in Table 2 and Supplementary Table 5

The hypothesized model (Fig. 1) tested the indirect relationships between maternal perceptions of child body mass (T1) and child BMI z-score (T2) via two sets of sequential mediators: (1) four maternal practices (T1) and (2) child screen use and physical activity (T2). These two sets of mediators were assumed to operate sequentially. The hypothesized model calculated for the total sample (*N* = 727 dyads) yielded an acceptable model-data fit, χ^2^ (52) = 147.112, *p* < .001, χ^2^/df = 2.829, GFI = .975, TLI = .924, NFI = .951, CFI = .967, RMSEA = .050 (90% CI: .041, .060). The variables included in the model accounted for 20.6% of the variance in child screen use (T2), 6.7% of the variance in child physical activity (T2) and 88.5% of the variance in child BMI z-score (T2), after adjustments were made for the effect of BMI z-score at T1. The unstandardized path coefficients and covariance coefficients are reported in Table 2.

**Table 2:** The hypothesized model: path and covariance coefficients for the study variables of the total sample (N = 729 dyads)

Variable	Hypothesized model with control variables			Hypothesized model without control variables		
Path coefficients/covariance coefficients	Unstandardized estimate	*SE*	*p* value	Unstandardized Estimate	*SE*	*p* value
*Associations between maternal perceptions of child body mass status (the independent variable; T1) and four maternal practices (the first set of mediators; T1)*						
Perceptions of child body mass (M, T1) → Restrictions of screen use (M, T1)	0.073	0.049	.138	0.067	0.050	.177
Perceptions of child body mass (M, T1) → Stimulation to be active (M, T1)	0.068	0.042	.105	0.065	0.042	.123
Perceptions of child body mass (M, T1) → Monitoring of screen use (M, T1)	0.036	0.061	.555	0.047	0.063	.457
Perceptions of child body mass (M, T1) → Monitoring of PA (M, T1)	− 0.016	0.044	.705	− 0.009	0.044	.846
*Associations between four maternal practices (the first set of mediators; T1), child screen use, and physical activity (the second set of mediators; T2)*						
Perceptions of child body mass (M, T1) → Screen use (Ch, T2)	**0.398**	**0.165**	**.016**	0.280	0.184	.129
Perceptions of child body mass (M, T1) → Physical activity (Ch, T2)	− 0.049	1.714	.977	− 0.050	1.754	.977
Restrictions of screen use (M, T1) → Screen use (Ch, T2)	0.025	0.151	.870	0.142	0.167	.396
Restrictions of screen use (Ch, T1) → Physical activity (Ch, T2)	0.541	1.571	.731	0.632	1.596	.692
Stimulation to be active (M, T1) → Screen use (Ch, T2)	**0.506**	**0.176**	**.004**	**0.670**	**0.196**	**< .001**
Stimulation to be active (M, T1) → Physical activity (Ch, T2)	**7.083**	**1.830**	** < .001**	**8.352**	**1.865**	**< .001**
Monitoring of screen use (M, T1) → Screen use (Ch, T2)	− 0.168	0.123	.171	− 0.180	0.136	.186
Monitoring of screen use (M, T1) → Physical activity (Ch, T2)	− **3.202**	**1.276**	**.012**	− **3.681**	**1.298**	**.005**
Monitoring of PA (M, T1) → Screen use (Ch, T2)	0.075	0.165	.649	− 0.042	0.184	.821
Monitoring of PA (M, T1) → Physical activity (Ch, T2)	1.695	1.718	.324	1.741	1.753	.321
*Associations between maternal perceptions of child body mass status, (the independent variable; T1), maternal practices, child behaviors (the mediators; T1 and T2), and child BMI z*-*score (the dependent variable; T2)*						
Perceptions of child body mass (M, T1) → BMI z-score (Ch, T2)	**0.067**	**0.033**	**.042**	**0.073**	**0.028**	**.010**
Restrictions of screen use (M, T1) → BMI z-score (Ch, T2)	− 0.034	0.026	.193	− 0.034	0.026	.192
Stimulation to be active (M, T1) → BMI z-score (Ch, T2)	− 0.018	0.031	.546	− 0.019	0.031	.546
Monitoring of screen use (M, T1) → BMI z-score (Ch, T2)	0.006	0.021	.777	0.010	0.021	.635
Monitoring of PA (M, T1) → BMI z-score (Ch, T2)	0.040	0.028	.156	0.045	0.028	.113
Screen use (Ch, T2) → BMI z-score (Ch, T2)	− 0.002	0.006	.705	− 0.004	0.006	.522
Physical activity (Ch, T2) → BMI z-score (Ch, T2)	− **0.002**	**0.001**	**.002**	− **0.002**	**0.001**	**.002**
BMI z-score (Ch, T1) → BMI z-score (Ch, T2)	**0.920**	**0.015**	** < .001**	**0.918**	**0.013**	**< .001**

*M* mother, *Ch* child, *T1* time 1 (baseline), *T2* time 2 (7- to 8-month follow-up), *PA* physical activity; perceptions of child body mass = maternal perceptions of child body mass status; restrictions of screen use = maternal restrictions of sedentary screen use behaviors; screen use = sedentary screen use behaviors; SES = perceived maternal economic status. Significant coefficients are marked in bold. The model without control variables included only one covariate, child BMI z-score at T1. The hypothesized model with control variables accounted for: T1 child screen use, T1 child physical activity, T1 child BMI-z score, age and gender of the child, maternal age, education, and perceived economic status at T1

Contrary to our hypothesis, there were no significant indirect effects of maternal perceptions of child body mass (T1) on child BMI z-score (T2) through the two sets of sequential mediators (four maternal practices and two child behaviors). Additional analyses were conducted to test whether maternal perceptions of child body mass (T1) and child BMI z-score (T2) may be indirectly related if only two sequential mediators are considered (e.g., maternal restrictions as the first mediator and child screen use as the second mediator). Eight nested models (four practices * two behaviors) were fit to investigate the effects of each maternal practice separately. No significant indirect effects were found (Supplement 1).

In line with the second hypothesis, indirect effects of two out of four maternal practices (T1) on child BMI z-score (T2) were found. Higher levels of monitoring of screen use (T1) were indirectly related to higher BMI z-score in children (T2) via lower levels of child physical activity (T2) acting as the mediator, *B* = 0.006, 90% BCI [0.001, 0.016]. Furthermore, higher maternal stimulation to be active (T1) was indirectly related to lower BMI z-score in children (T2) via higher levels of child physical activity (T2), *B* = −  0.014, 90% BCI [− 0.034, − 0.002]. There were no other significant indirect effects.

Results of the sensitivity analysis, testing the hypothesized model accounting for T1 child BMI z-score as the control variable but without other control variables, indicated the same patterns of associations as in the model with eight control variables, except for a direct association between maternal perceptions of child body mass (T1) and child screen use (T2). This association was not significant in the model without the control variables.

## Discussion

This study unravels complex associations between maternal perceptions of child body mass status, four maternal practices related to child screen use and physical activity, child screen use, child physical activity, and child BMI z-score. Two maternal practices indirectly predicted child BMI z-score at the follow-up while adjustments were made for the BMI z-score at baseline. Maternal reports of stimulating their children to be active were indirectly related to lower child BMI z-score via an increase in child physical activity. In contrast, maternal reports of frequent monitoring of screen use were indirectly associated with higher child BMI z-score via lower levels of child physical activity.

Direct comparisons between the results of the present study and previous research are difficult due to shortcomings of the research designs of previous studies (child self-report only or parental report only; Gubbels et al., 2011, Sleddens et al., 2017; or cross-sectional study designs, e.g., Lloyd et al., 2014). Importantly, previous research did not show consistent patterns of associations between parental practices, child energy expenditure behaviors, and child body mass developments (Gubbels et al., 2011, Lloyd et al., 2014, Sleddens et al., 2017). To the best of our knowledge, this is the first study to overcome these design-related limitations. Across the analyzed maternal strategies, stimulation to be active was related to healthy body mass development in children, mediated by a high level of physical activity. Stimulation is operationalized as encouragement to engage in physical activity (Sleddens et al., 2017) and is thus operationalized in a way that may be similar to social support for PA (Edwardson & Gorely, 2010). Systematic reviews (Edwardson & Goreley, 2010) showed that PA encouragement is one of the best predictors of child physical activity (yet, the accumulated evidence was mostly gathered from cross-sectional research). The present study did not confirm the beneficial effects of maternal restrictions and monitoring on healthy body mass development. Restrictions are considered a control-based strategy (Liszewska, Scholz, Radtke, Horodyska, & Luszczynska, 2018), with monitoring being closely related to control-based strategies (it is assessed as the extent to which mothers oversee the behavior of their children; Sleddens et al., 2017). In the context of energy intake behaviors, parental use of control-based strategies has been shown to form nonsignificant or positive associations with child body mass (Liszewska et al., 2018).

On the other hand, we found that in addition to being related to higher levels of physical activity in children, maternal stimulation to be active (T1) was also directly related to a higher level of screen use in children (T2). First, this effect may occur due to child reactance and tendencies to act (at least in part) against parental recommendations (Johnson & Buboltz, 2000). Child reactance may, in turn, depend on factors such as relationship quality. Previous dyadic research has indicated that relationship quality may directly predict the physical activity of dyadic partners (Knoll et al., 2017). Future research may investigate whether relationship quality moderates the effects of parental strategies on child behaviors. Second, the effect may be explained as matching some of the assumptions made by a compensatory approach to health behaviors (Knäuper et al., 2004). According to this approach, people believe that if they perform a health-promoting behavior (e.g., physical activity), they can reward themselves with an unhealthy behavior (e.g., screen use). It is possible that children who engaged in high levels of physical activity (after being exposed to high levels of maternal stimulation to be active) may compensate for their physical activity by engaging in sedentary behaviors, such as screen use. Thus, maternal stimulation to be active may also have a compensatory effect on behavior, and it may result in more time spent on screen use. To further clarify the links between parental stimulation, child physical activity, and screen use, children’s compensatory beliefs (their reasons for engaging in physical activity and screen use) should be evaluated. Finally, the actual content of the actions representing maternal stimulation to be active might determine the effects of maternal strategies on screen use in children. For example, if the stimulation indicates a reward for engagement in physical activity (such as playing a favorite screen-based game), this could indeed promote both physical activity and sedentary behaviors in children. Future search needs to account for the actual content and the sequence of maternal practices, including the use of rewards for an increase in physical activity.

The second significant indirect effect found in the present study suggested that low levels of maternal reports of monitoring screen use (T1) were related to higher physical activity in children and, in turn, healthy body mass developments. These findings may be interpreted as partially in line with those of a longitudinal study conducted in the context of food-related strategies that showed that low levels of control-based parental strategies were linked with healthier child BMI z-score (Liszewska et al., 2018). Although monitoring is not a typical control-based strategy, its core refers to parental overseeing of child behavior.

Although previous studies have yielded evidence for associations between maternal perceptions of child body mass status and maternal nutrition-related practices (Merema et al., 2015; Sylvetsky-Meni, Gillepsie, Hardy, & Welsh, 2015; Wehrly et al., 2014; Yilmaz et al., 2013), the present study did not support such associations for the physical activity context. This may be due to the study’s focus on maternal strategies referring to physical activity and screen use, whereas previous research focused mainly on maternal strategies referring to nutrition. Future research should clarify whether the effects of parental strategies are specific to target behaviors.

The present study indicated that screen use and physical activity were unrelated. These findings contribute to the discussion on the distinct character of sedentary behavior and physical activity (van der Ploeg & Hillsdon, 2017). Sedentary screen use and physical activity may form two independent behaviors (van der Ploeg & Hillsdon, 2017). The strongest determinants of screen use may differ from the strongest determinants of physical activity. The availability of screen-based equipment at home may be the key determinant of screen use (Boberska et al., 2019), whereas physical environment characteristics, including available sport facilities, accessible parks, or cycling trails, may be the key determinants of physical activity (Sallis et al., 2006). Future research should control for the effects of environmental determinants specific for sedentary behavior and for physical activity.

In addition to many strengths, the present study also has certain limitations. Preferable measurements of screen use and physical activity combine self-reports with accelerometers, including posture data (Montoye, Pivarnik, Mudd, Biswas, & Pfeiffer, 2017). The measurement of maternal practices used in the present study might also have captured a broader range of strategies representing parental control. Screen use was measured with only two items, which assessed various screen-related behaviors (TV-viewing, computer games, tablet use). The questions about watching video channels or smartphone use were not included. The applied measure of physical activity had relatively low reliability. Problems with understanding questions as written and poor recall may be among the major reasons of low reliability of physical activity assessment in young children. Additionally, low reliability may be related to the content of the items that could be adjusted to fit young children’s physical activity. The limited fit of the content of the items with typical sports played by young children could also reduce the validity of the measure. Accelerometer-based measures of sedentary behavior (including screen use) and physical activity would be recommended to obtain more reliable and valid data. The conclusions drawn from the present study should be considered preliminary until the obtained patterns of associations are replicated in research using accelerometer-based data. Finally, the study did not address food intake, sleep, or other factors such as stress and anxiety, which can have an important role affecting child body mass and/or child physical activity.

The present findings may have implications for practice. In line with previous research (Cislak, Safron, Pratt, Gaspar, & Luszczynska, 2012), our findings suggest that mothers may be encouraged to stimulate their children to be active. Additionally, mothers should also be aware that high monitoring of screen use may be associated with lower physical activity in children. When developing obesity prevention interventions, practitioners may take into account that maternal practices aiming at child physical activity may be unrelated to maternal perceptions of their child body mass status. Future studies may test whether the obtained patterns of associations would be similar across subgroups (e.g., father-son vs mother-son dyads).

In conclusion, the findings shed light on complexities in the relationships between maternal perceptions of child weight status, four maternal practices, child screen use behaviors, physical activity, and child BMI z-score. Two maternal practices were indirectly linked with child BMI z-score. High stimulation to be active and low monitoring of screen use were related to lower BMI z-score in children (assessed 7- to 8-months later) via physical activity in children.

## Electronic supplementary material

Below is the link to the electronic supplementary material.

Supplementary material 1 (DOCX 62 kb)

## Data Availability

The respective dataset is available at https://osf.io/r8qmg/.
