# Supplementary material for: Maternal practices and perceptions of child body mass status explain child energy expenditure behaviors and body mass
Source: J Behav Med. 2020 Jan 31;43(6):904–15. doi: 10.1007/s10865-020-00138-1 (PMC7674564; doi:10.1007/s10865-020-00138-1)
Supplement: Supplementary file 1 — Supplementary material 1 (DOCX 62 kb) [file 10865_2020_138_MOESM1_ESM.docx]

**Maternal practices and perceptions of child body mass status explain child energy expenditure behaviors and body mass**

**Supplement 1**

**Sample Characteristics: Mothers’ Education, Employment, Perceived Economic Status, and Ethnicity**

The majority of mothers had either secondary education (27.6%) or higher education (40.2%), 14.4% had vocational education and 12.2% post-secondary education, whereas 5.6% reported to have primary education only. Fifty-nine percent of mothers reported full-time employment, 15.5% were employed part-time, and 25.5% reported no current employment or being retired. More than half of the mothers (59%) reported that their perceived economic status was similar to the economic status of the average family in Poland, with 31% indicating that their economic status was higher, and the remaining 10% evaluated their economic status as lower. The majority of mothers (68%) lived in urban areas, whereas the remaining mothers lived in rural areas. All participants were white (62% of Poland’s population lives in urban areas, 98% of population is white, see Demographic Yearbook of Poland, 2015).

**Results of Attrition Analysis**

Attrition analysis indicated that mothers who completed T1 and T2 did not differ from those who dropped out in terms of: restrictions of screen use, *F*(1, 727) = 0.77, *p* = .380, stimulation to be active, *F*(1, 727) = 0.03, *p* = .871, monitoring of screen use, *F*(1, 727) = 0.95, *p* = .329, monitoring of PA, *F*(1, 727) = 0.25, *p* = .619, perceived economic status, *F*(1, 727) = 0.33, *p* = .566, and education, *χ*^2^(4) = 6.43, *p* = .169. However, there were differences in maternal age, *F*(1, 727) = 4.47, *p* = .03, *η*² = .006 (completers: *M* = 36.40, *SD* = 5.74; dropouts: *M* = 35.46, *SD* = 5.35). Children who completed both T1 and T2 did not differ from children who dropped out in terms of age, *F*(1, 727) = 0.82, *p* = .365, gender, *χ*^2^(1) = 0.37, *p* = .541, and time spent on physical activity, *F*(1, 727) = 0.00, *p* = .995. There were differences in screen use, *F*(1, 727) = 13.51, *p* < .001, *η*² = .018 (completers: *M* = 3.11, *SD* = 2.71; dropouts: *M* = 4.01, *SD* = 3.74), and child BMI z-score, *F*(1,727) = 9.77, *p* = .002, *η*² = .013 (completers: *M* = 0.35, *SD* = 1.18, dropouts: *M* = 0.66, *SD* = 1.32).

**Differences Between Mother-Daughter and Mother-Son Dyads**

Depending on child’s gender, analyses indicated differences in maternal practices: restrictions of screen use, *F*(1, 727) = 14.46, *p* < .001, *η*^2^ = .140, with mothers restricting boys more often (*M* = 2.86, *SD* = 0.67) than girls (*M* = 2.66, *SD* = 0.77); maternal monitoring of screen use, *F*(1, 727) = 5.57, *p* = .019, *η*^2^ = .087, with more monitoring among boys (*M* = 4.10 *SD* = 0.87) than girls (*M* = 3.94, *SD* = 0.98); stimulation to be active, *F*(1, 727) = 9.22, *p* = .002, *η*^2^ = .011, with more stimulation among boys (*M* = 3.18 *SD* = 0.61) than girls (*M* = 3.04, *SD* = 0.63). There were also significant differences in physical activity, *F*(1, 727) = 5.58, *p* = .018, *η*^2^ = .087, with boys (*M* = 59.9, *SD* = 28.34) being more physically active than girls (*M* = 55.3 *SD* = 24.63); and screen use, *F*(1, 727) = 16.03, *p* < .001, *η*^2^ = .141, with boys reporting more screen use (*M* = 3.64 *SD* = 3.24) than girls (*M* = 2.86, *SD* = 1.94).

**Differences Between Dyads with Younger and Older Children (Supplementary Table 1)**

Differences between dyads with younger children (≤ 8 years old) and dyads with older children (> 8 years old) are presented in Supplementary Table 1. Overall, the majority of differences were not significant, except for child BMI z-score and maternal age.

**Maternal Perceptions of Child Body Mass by Child Body Mass Status and by Meeting Physical Activity Recommendations by Children (Supplementary Tables 2 and 3)**

Next, Supplement 1 reports the percent of child body mass levels and maternal perceptions of child body mass. The differences between children with overweight, normal weight, and underweight are presented in Supplementary Table 2. Further, Supplementary Table 3 presents the how often children with overweight, normal weight, and underweight were meeting physical activity recommendations and how often the children were perceived by mothers as having overweight, normal weight, or underweight. Overall, the majority of mothers considered that their child had normal body mass, regardless of the actual body mass status of their children. Only 0.7% of children met physical activity recommendations (WHO, 2018).

**The Moderating Effects of Age Group and Child Body Mass Status on the Hypothesized Mediation Model**

Additional analyses were conducted to test the hypothesized effects in two-group models: (1) dyads with children ≤ 8 years old vs. dyads with children > 8 years old, and (2) dyads with children with normal body mass vs dyads with children with excessive body mass. In general, the analyses indicated no significant differences between the two age groups, nor between the two groups with normal vs. excessive body mass.

**Child Age.** It was tested whether the results obtained for the hypothesized model would differ in dyads with children ≤ 8 years old, compared to dyads with children > 8 years old. To investigate these between-group differences, the unconstrained model was compared to the constrained nested model, assuming that the respective paths are equal across the group of dyads with children ≤ 8 years old vs. the group of dyads with children > 8 years old: (1) from maternal perceptions of child body mass (T1) to maternal practices (restriction of screen use, stimulation to be active, monitoring of screen use, monitoring of PA) (T1), (2) from maternal practices (T1) to child behavior (screen use, physical activity) (T2), and (3) from child behavior (T2) to child BMI z-score (T2).

The constrained model-data fit was acceptable, *χ*^2^(127) = 202.534, *p* < .001, *χ*^2^/df = 1.792, GFI = .964, NFI = .935, TLI = .943, CFI = .969, RMSEA = .033 (90% CI: .026, .040). The hypothesized unconstrained model and the constrained nested model did not differ significantly, Δ*χ*^2^ = 28.000, df = 21, *p* = .140, ΔTLI = - .008. A lack of significant differences between the hypothesized unconstrained model and the nested constrained model may be interpreted as indicating similarity in the overall patterns of the associations in dyads with children ≤ 8 years old, compared to dyads with children > 8 years old.

**Child Body Mass Status.** It was also tested whether the hypothesized associations would differ in dyads with normal body mass vs. dyads with excessive body mass children. To investigate the between-group differences, the unconstrained model was compared to the nested constrained model, assuming that the respective paths are equal across the group of dyads with children with normal body mass vs the group of dyads with children with excessive body mass: (1) from maternal perceptions of child body mass (T1) to maternal practices (restriction of screen use, stimulation to be active, monitoring of screen use, monitoring of PA) (T1), (2) from maternal practices (T1) to child behavior (screen use, physical activity) (T2), and (3) from child behavior (T2) to child body mass (T2).

The constrained model-data fit was acceptable, *χ*^2^(141) = 155.311, *p* < .001, *χ*^2^/df = 1.569, GFI = .970, NFI = .882, TLI = .892, CFI = .949, RMSEA = .030 (90% CI: .020, .038). The hypothesized unconstrained model and the constrained nested model did not differ significantly, Δ*χ*^2^ = 0.732, df = 21, *p* = .803, ΔTLI = - .043. A lack of significant differences between the hypothesized unconstrained model and the nested constrained model indicate a similar pattern of the associations in dyads with children with normal body mass, compared to dyads with children with excessive body mass.

**Findings for the Mediating Effects of Each of the Maternal Practices Operating Separately (Supplementary Table 4)**

Additional analyses were conducted to test if maternal perceptions of child body mass (T1) and child BMI z-score (T2), may be indirectly related if only two sequential mediators are considered (e.g., maternal restrictions and child screen use). Eight nested models (four practices * two behaviors) were calculated to investigate the effects of each maternal practice separately. This step was included because even if the overall indirect effect, composed of parallel mediators operating simultaneously, is not significant, a significant indirect effect may exist for a model assuming only two sequential mediators.

Therefore, the sequential two-mediator nested models were tested (e.g., maternal perceptions of child body mass 🡪 maternal restrictions of screen use 🡪 child screen use 🡪 child BMI z-score). The indirect effects of each of the sequential two-mediator nested models were tested assuming that besides the tested indirect paths, all other indirect paths (to and from the mediators) are constrained to zero (for a similar approach see Zarychta et al., 2019).

The indirect effects for the hypothesized unconstrained models (with and without additional covariates) and eight two-mediator nested models are presented in Supplementary Table 4. In line with the findings obtained for the hypothesized model, the sequential two-mediator nested models yielded no significant indirect effects of maternal perceptions of child body mass on child BMI z-score.

**Effects of Covariates in the Hypothesized Mediation Model (Supplementary Table 5)**

Supplementary Table 5 presents the effects of all covariates and covariances between the main variables in the hypothesized model.

**References**

Zarychta, K., Horodyska, K., Gan, Y., Chan, C. K. Y., Wiggers, J., Wolfenden, L., . . . Luszczynska, A. (2019). Associations of parental and child food and exercise aversion with child food intake and physical activity. *Health Psychology*, *38*(12), 1116-1127. http://dx.doi.org/10.1037/hea0000799

Supplementary Table 1

*Differences in the study variables between dyads with younger children (aged ≤ 8-years-old, n = 261, 35.8%) and older children (aged > 8-years-old, n = 468, 64.2%) in N = 729 mother-child dyads.*

|  | *Differences between dyads with children ≤ 8 years old vs dyads with children > 8 years old*  *F (df)* | *M (SD) in dyads with children ≤ 8 years old /*  *M (SD) in dyads with children > 8 years old* | *η^2^* | *Cohen’s d (95% CI)* |
| --- | --- | --- | --- | --- |
| 1. Perceptions of child body mass (M, T1) | 0.14 (1,728) | 2.99 (0.41) / 3.01 (0.62) | < 0.001 | - 0.04 (- 0.08, 0.01) |
| 2. Restrictions of screen use (M, T1) | 1.45 (1,728) | 2.80 (0.72) / 2.73 (0.75) | 0.002 | 0.10 (0.04, 0.15) |
| 3. Monitoring of screen use (M, T1) | **9.11 (1,728)**** | **4.16 (0.85) / 3.94 (0.96)** | **0.012** | **0.24 (0.17, 0.31)** |
| 4. Monitoring of PA (M, T1) | 2.16 (1,728) | 3.42 (0.60) / 3.35 (0.68) | 0.003 | 0.11 (0.06, 0.16) |
| 5. Stimulation to be active (M, T1) | 0.30 (1,728) | 3.12 (0.60) / 3.09 (0.64) | < 0.001 | 0.05 (0.01, 0.09) |
| 6. Screen use (Ch, T1) | 0.21 (1,728) | 3.47 (3.32) / 3.36 (2.98) | < 0.001 | 0.04 (- 0.19, 0.26) |
| 7. Screen use (Ch, T2) | < 0.01 (1,728) | 3.33 (2.66) / 3.34 (2.82) | < 0.001 | - 0.01 (- 0.20, 0.20) |
| 8. Physical activity (Ch, T1) | 0.91 (1,728) | 54.19 (28.76) / 56.40 (30.46) | 0.001 | - 0.07 (- 2.24, 2.09) |
| 9. Physical activity (Ch, T2) | 5.20 (1,728)* | 54.48 (24.37) / 59.14 (27.54) | 0.007 | - 0.18 (- 2.09, 1.74) |
| 10. BMI z-score (Ch, T1) | **12.11 (1,728)**** | **0.23 (1.21) / 0.56 (1.24)** | **0.016** | **- 0.27 (- 0.36, - 0.18)** |
| 11. BMI z-score (Ch, T2) | **9.15 (1,728)**** | **0.13 (1.21) / 0.42 (1.22)** | **0.012** | **- 0.24 (- 0.33, - 0.15)** |
| 12. Education (P, T1) | 0.98 (1,728) | 3.73 (1.24) / 3.63 (1.31) | 0.001 | 0.08 (- 0.02, 0.17) |
| 13. SES (M, T1) | 0.71 (1,728) | 2.71 (0.80) / 2.76 (0.80) | 0.001 | - 0.06 (- 0.12, - 0.01) |
| 14. Age (M, T1) | **12.66 (1,728)***** | **35.12 (4.97) / 36.66 (5.96)** | **0.017** | **- 0.27 (- 0.68, 0.13)** |
| 15. Gender (Ch, T1) | 2.14 (1,728) | 1.57 (0.50) / 1.51 (0.50) | 0.003 | 0.12 (0.08, 0.16) |

*Note.* ****p* < .001; ***p* < .01; **p* < .05. M = mother; Ch = child; T1 = time 1 (the baseline); T2 = time 2 (the 7-to 8-month follow-up); PA = physical activity; perceptions of child body mass = maternal perceptions of child body mass status; restrictions of screen use = maternal restrictions of sedentary screen use behaviors; screen use = sedentary screen use behaviors; SES = maternal perceived economic status. Significant differences (with significant *p*-levels and significant 95% CI for Cohen’s *d*) are marked in bold.

Supplementary Table 2

*The percent of children with underweight, normal, overweight/obesity and maternal perceptions of child body mass (N = 729 mother-child dyads).*

| *Child body mass status (underweight, normal, overweight/obese), measured objectively*  *n*  *(%)* | | *Percent of children perceived by mothers as overweight/obese (12.8% for the total sample), normal body mass (74.3%), and underweight (12.9%) across children with overweight/obesity, normal weight, and underweight*  *n  (%)* | | *A comparison of*  *children underweight, normal, overweight/obesity*  *and maternal perceptions of child body mass* | *χ*^2^ (df) | η^2^ |
| --- | --- | --- | --- | --- | --- | --- |
| Overweight/obese | 172  (23.6%) | Overweight/obese | 72  (41.9%) | A comparison of overweight/obese children vs children with normal body mass in terms of maternal perception of child body mass | 151.31  (1, 729)  *** | 0.230 |
|  |  | Normal | 91  (52.9%) |  |  |  |
|  |  | Underweight | 9  (5.2%) |  |  |  |
| Normal | 485  (66.5%) | Overweight/obese | 20  (4.1%) | A comparison of overweight/obese children vs underweight children in terms of maternal perception of child body mass | 70.59  (1, 729)  *** | 0.289 |
|  |  | Normal | 402  (82.9%) |  |  |  |
|  |  | Underweight | 63  (13.0%) |  |  |  |
| Underweight | 72  (9.9%) | Overweight/obese | 0  (0.0%) | A comparison of children with normal body mass vs underweight children and maternal perception of child body mass | 35.44  (1, 729)  *** | 0.064 |
|  |  | Normal | 43  (59.7%) |  |  |  |
|  |  | Underweight | 29  (40.3%) |  |  |  |

*Note.* ****p* < .001.

Supplementary Table 3

*Meeting physical activity recommendations across the subgroups of (1) children with overweight/obesity, normal body mass, and underweight, and (2) children perceived by their mothers as overweight/obese, with normal body mass, and underweight. Findings from (N = 729 mother-child dyads).*

| *Child body mass status (underweight, normal, overweight/obese)*  *n*  *(%)* | | *Meeting physical activity recommendations in children with overweight/obesity, normal body mass, and underweight*  *n*  *(%)* | | *Maternal perception of child body mass (underweight, normal, overweight/obese)*  *n*  *(%)* | | *Meeting physical activity recommendations in children perceived by their mothers as overweight/obese, normal weight, and underweight*  *n*  *(%)* | |
| --- | --- | --- | --- | --- | --- | --- | --- |
| Overweight/obese | 172  (23.6%) | Meeting PA recommendations | 1  (0.6%) | Overweight / obesity | 92  (12.6%) | Meeting PA recommendations | 1  (1.1%) |
|  |  | Not meeting PA recommendations | 171  (99.4%) |  |  | Not meeting PA recommendations | 91  (98.9%) |
| Normal | 485  (66.5%) | Meeting PA recommendations | 4  (0.8%) | Normal weight | 536  (73.5%) | Meeting PA recommendations | 4  (0.7%) |
|  |  | Not meeting PA recommendations | 481  (99.2%) |  |  | Not meeting PA recommendations | 532  (99.3%) |
| Underweight | 72  (9.9%) | Meeting PA recommendations | 0  (0.0%) | Underweight | 101  (13.9%) | Meeting PA recommendations | 0  (0.0%) |
|  |  | Not meeting PA recommendations | 72  (100.0%) |  |  | Not meeting PA recommendations | 101  (100.0%) |

*Note.* PA = physical activity. PA thresholds were based on WHO recommendations (2018), suggesting that children should perform at least 60 minutes of moderate-to-vigorous physical activity daily.

Supplementary Table 4

*Indirect effects for the hypothesized unconstrained models (with and without additional covariates) and nested models.*

| The model:  assumed indirect pathways | Model-data fit indices | | | | | | Indirect effects^b^ | | |
| --- | --- | --- | --- | --- | --- | --- | --- | --- | --- |
|  | *χ^2^*(df)^a^ | *χ^2^*/df | NFI | TLI | CFI | RMSEA (95% CI) | *B* | *SE* | 95% CI |
| *The hypothesized unconstrained models* |  |  |  |  |  |  |  |  |  |
| The hypothesized unconstrained model with covariates:  perceptions of child body mass (M, T1) → mediators (restriction of screen use [M, T1], stimulation to be active [M, T1], monitoring of screen use [M, T1], monitoring of PA [M, T1]) → mediators (screen use [Ch, T2], physical activity [Ch, T2]) → BMI z-score (Ch, T2) | 108.78 (52) | 2.09 | .945 | .929 | .969 | .048  (.036, .061) | - 0.013 | 0.014 | - 0.055, 0.003 |
| The hypothesized unconstrained model without covariates (except for BMI z-score at T1):  perceptions of child body mass (M, T1) → mediators (restriction of screen use [M, T1], stimulation to be active [M, T1], monitoring of screen use [M, T1], monitoring of PA [M, T1]) → mediators (screen use [Ch, T2], physical activity [Ch, T2]) → BMI z-score (Ch, T2) | 226.80 (7) | 32.40 | .911 | .548 | .912 | .208  (.185, .231) | - 0.005 | 0.008 | - 0.027, 0.006 |
| *The nested models^c^* |  |  |  |  |  |  |  |  |  |
| 1^st^ nested model:  perceptions of child body mass (M, T1) → restrictions of screen use (M, T1) → screen use (Ch, T2) → BMI z-score (Ch, T2) | 149.24 (70) | 2.13 | .924 | .926 | .957 | .049  (.038, .060) | < - 0.001 | 0.001 | - 0.004, < 0.001 |
| 2^nd^ nested model:  perceptions of child body mass (M, T1) → restrictions of screen use (M, T1) → physical activity (Ch, T2) → BMI z-score (Ch, T2) | 147.59 (70) | 2.11 | .925 | .928 | .958 | .049  (.038, 060) | < - 0.001 | < 0.001 | - 0.002, < 0.001 |
| 3^rd^ nested model:  perceptions of child body mass (M, T1) → stimulation to be active (M, T1) → screen use (Ch, T2) → BMI z-score (Ch, T2) | 144.58 (70) | 2.07 | .926 | .931 | .960 | .048  (.037, .059) | < - 0.001 | 0.001 | - 0.004, < 0.001 |
| 4^th^ nested model:  perceptions of child body mass (M, T1) → stimulation to be active (M, T1) → physical activity (Ch, T2) → BMI z-score (Ch, T2) | 140.25 (70) | 2.00 | .929 | .935 | .962 | .046  (.035, .058) | < - 0.001 | 0.001 | - 0.003, < 0.001 |
| 5^th^ nested model:  perceptions of child body mass (M, T1) → monitoring of screen use (M, T1) → screen use (Ch, T2) → BMI z-score (Ch, T2) | 152.43 (70) | 2.18 | .922 | .923 | .955 | .050  (.039, .061) | < - 0.001 | < 0.001 | - 0.001, < 0.001 |
| 6^th^ nested model:  perceptions of child body mass (M, T1) → monitoring of screen use (M, T1) → physical activity (Ch, T2) → BMI z-score (Ch, T2) | 150.19 (70) | 2.15 | .924 | .925 | .957 | .050  (.039, .060) | < 0.001 | < 0.001 | < -0.001, 0.001 |
| 7^th^ nested model:  perceptions of child body mass (M, T1) → monitoring of PA (M, T1) → screen use (Ch, T2) → BMI z-score (Ch, T2) | 151.21 (70) | 2.16 | .923 | .925 | .956 | .050  (.039, .061) | < 0.001 | < 0.001 | < 0.001, 0.002 |
| 8^th^ nested model:  perceptions of child body mass (M, T1) → monitoring of PA (M, T1) → physical activity (Ch, T2) → BMI z-score (Ch, T2) | 145.58 (70) | 2.08 | .926 | .930 | .959 | .048  (.037, .059) | < 0.001 | < 0.001 | < 0.001, 0.002 |

*Note.* M = mother; Ch = child; T1 = time 1 (the baseline); T2 = time 2 (the 7- to 8-month follow-up); PA = physical activity; perceptions of child body mass = maternal perceptions of child body mass status; restrictions of screen use = maternal restrictions of sedentary screen use behaviors; screen use = sedentary screen use behaviors.

Supplementary Table 5.

*The path coefficients (the effects of the controlled variables on the main study variables) and covariance coefficients among the study variables in the hypothesized model (N = dyads 729). For the direct associations between the main variables of the study see the main manuscript, Table 2.*

| Variable | Hypothesized model with control variables | | | Hypothesized model without control variables | | |
| --- | --- | --- | --- | --- | --- | --- |
| Path coefficients/covariance coefficients | Unstandardized estimate | *SE* | *p*-value | Unstandardized estimate | *SE* | *p*-value |
| **Predictors of four maternal practices (T1)** |  |  |  |  |  |  |
| Age (Ch, T1) 🡪 Restrictions of screen use (M, T1) | - 0.029 | 0.020 | .160 |  |  |  |
| Age (Ch, T1) 🡪 Stimulation to be active (M, T1) | - 0.022 | 0.017 | .205 |  |  |  |
| Age (Ch, T1) 🡪 Monitoring of screen use (M, T1) | **- 0.069** | **0.025** | **.007** |  |  |  |
| Age (Ch, T1) 🡪 Monitoring of PA (M, T1) | - 0.025 | 0.018 | .174 |  |  |  |
| Age (M, T1) 🡪 Restrictions of screen use (M, T1) | 0.001 | 0.005 | .907 |  |  |  |
| Age (M, T1) 🡪 Stimulation to be active (M, T1) | 0.006 | 0.004 | .142 |  |  |  |
| Age (M, T1) 🡪 Monitoring of screen use (M, T1) | 0.001 | 0.006 | .864 |  |  |  |
| Age (M, T1) 🡪 Monitoring of PA (M, T1) | < 0.001 | 0.004 | .933 |  |  |  |
| Gender (Ch, T1) 🡪 Restrictions of screen use (M, T1) | **- 0.208** | **0.054** | **< .001** |  |  |  |
| Gender (Ch, T1) 🡪 Stimulation to be active (M, T1) | **- 0.139** | **0.046** | **.002** |  |  |  |
| Gender (Ch, T1) 🡪 Monitoring of screen use (M, T1) | **- 0.151** | **0.068** | **.025** |  |  |  |
| Gender (Ch, T1) 🡪 Monitoring of PA (M, T1) | - 0.044 | 0.048 | .359 |  |  |  |
| Education (M, T1) 🡪 Restrictions of screen use (M, T1) | 0.018 | 0.022 | .423 |  |  |  |
| Education (M, T1) 🡪 Stimulation to be active (M, T1) | 0.017 | 0.019 | .369 |  |  |  |
| Education (M, T1) 🡪 Monitoring of screen use (M, T1) | **0.083** | **0.028** | **.002** |  |  |  |
| Education (M, T1) 🡪 Monitoring of PA (M, T1) | **0.064** | **0.020** | **.001** |  |  |  |
| SES (M, T1) 🡪 Restrictions of screen use (M, T1) | 0.021 | 0.035 | .549 |  |  |  |
| SES (M, T1) 🡪 Stimulation to be active (M, T1) | 0.042 | 0.030 | .160 |  |  |  |
| SES (M, T1) 🡪 Monitoring of screen use (M, T1) | **0.099** | **0.044** | **.026** |  |  |  |
| SES (M, T1) 🡪 Monitoring of PA (M, T1) | 0.013 | 0.031 | .672 |  |  |  |
| **Predictors of child screen use (T2) and PA (T2)** |  |  |  |  |  |  |
| Age (Ch, T1) 🡪 Screen use (Ch, T2) | - 0.042 | 0.068 | .538 |  |  |  |
| Age (Ch, T1) 🡪 Physical activity (Ch, T2) | **1.867** | **0.704** | **.008** |  |  |  |
| Gender (Ch, T1) 🡪 Screen use (Ch, T2) | **- 0.564** | **0.185** | **.002** |  |  |  |
| Gender (Ch, T1) 🡪 Physical activity (Ch, T2) | - 2.784 | 1.918 | .147 |  |  |  |
| Screen use (Ch, T1) 🡪 Screen use (Ch, T2) | **0.370** | **0.029** | **< .001** |  |  |  |
| Physical activity (Ch, T1) 🡪 Physical activity (Ch, T2) | **0.139** | **0.032** | **< .001** |  |  |  |
| **Predictors of child BMI z-score (T2)** |  |  |  |  |  |  |
| Age (Ch, T1) 🡪 BMI z-score (Ch, T2) | - 0.007 | 0.012 | .550 |  |  |  |
| Age (M, T1) 🡪 BMI z-score (Ch, T2) | - 0.001 | 0.003 | .720 |  |  |  |
| Gender (Ch, T1) 🡪 BMI z-score (Ch, T2) | - 0.016 | 0.032 | .615 |  |  |  |
| Education (M, T1) 🡪 BMI z-score (Ch, T2) | **0.025** | **0.013** | **.050** |  |  |  |
| SES (M, T1) 🡪 BMI z-score (Ch, T2) | - 0.012 | 0.020 | .540 |  |  |  |
| **Covariances** |  |  |  |  |  |  |
| Perceptions of child body mass (M, T1) 🡨🡪 BMI z-score (Ch, T1) | **0.352** | **0.028** | **< .001** |  |  |  |
| Perceptions of child body mass (M, T1) 🡨🡪 Age (Ch, T1) | - 0.004 | 0.023 | .870 |  |  |  |
| Perceptions of child body mass (M, T1) 🡨🡪 Age (M, T1) | 0.033 | 0.098 | .738 |  |  |  |
| Perceptions of child body mass (M, T1) 🡨🡪 SES (M, T1) | 0.023 | 0.014 | .106 |  |  |  |
| Perceptions of child body mass (M, T1) 🡨🡪 Education (M, T1) | **0.072** | **0.023** | **.002** |  |  |  |
| SES (M, T1) 🡨🡪 Education (M, T1) | **0.297** | **0.040** | **< .001** |  |  |  |
| Age (Ch, T1) 🡨🡪 Age (M, T1) | **1.332** | **0.285** | **< .001** |  |  |  |
| Perceptions of child body mass (M, T1) 🡨🡪 Gender (Ch, T1) | **0.019** | **0.009** | **.034** |  |  |  |
| Restrictions of screen use (M, T1) 🡨🡪 Stimulation to be active (M, T1) | **0.194** | **0.018** | **< .001** | **0.204** | **0.019** | **< .001** |
| Restrictions of screen use (M, T1) 🡨🡪 Monitoring screen use (M, T1) | **0.333** | **0.027** | **< .001** | **0.350** | **0.029** | **< .001** |
| Restrictions of screen use (M, T1) 🡨🡪 Monitoring PA (M, T1) | **0.181** | **0.019** | **< .001** | **0.188** | **0.019** | **< .001** |
| Stimulation to be active (M, T1) 🡨🡪 Monitoring screen use (M, T1) | **0.248** | **0.023** | **< .001** | **0.263** | **0.024** | **< .001** |
| Stimulation to be active (M, T1) 🡨🡪 Monitoring PA (M, T1) | **0.178** | **0.016** | **< .001** | **0.184** | **0.017** | **< .001** |
| Monitoring SB (M, T1) 🡨🡪 Monitoring PA (M, T1) | **0.256** | **0.024** | **< .001** | **0.273** | **0.025** | **< .001** |
| Screen use (Ch, T2) 🡨🡪 Physical activity (Ch, T2) | 0.210 | 2.309 | .928 | 0.453 | 2.625 | .863 |

*Note.* M = mother; Ch = child; T1 = time 1 (the baseline); T2 = time 2 (the 7- to 8-month follow-up); PA = physical activity; perceptions of child body mass = maternal perceptions of child body mass status; restrictions of screen use = maternal restrictions of sedentary screen use behaviors; screen use = sedentary screen use behaviors; SES = maternal perceived economic status. Significant coefficients are marked in bold. The hypothesized model with control variables: T1 child screen use, T1 child PA, and T1 Child BMI-z score, child’s gender, maternal and child age, maternal education, and perceived economic status at T1. The model without control variables included child BMI z-score at T1 as the only controlled variable.
